# A case report of Trousseau syndrome

**DOI:** 10.1097/MD.0000000000034449

**Published:** 2023-07-28

**Authors:** Xiao Jing Liu, Yu Xiang Liu, Ning Yuan Zhang

**Affiliations:** a Department of Neurology, The First People’s Hospital of Tongxiang, Zhejiang, People’s Republic of China

**Keywords:** D-Dimer, ischemic stroke, Trousseau syndrome, TTS

## Abstract

In 1865, Trousseau first discovered pulmonary embolism caused by multiple venous thrombosis in patients with gastric cancer, and later all clinical manifestations of malignant patients during pathogenesis due to abnormal coagulation and fibrinolysis were referred to collectively as Trousseau syndrome. Trousseau syndrome is not a benign thrombophlebitis, and when diagnosed it requires immediate treatment. The survival rate over 1 year is only 12%. Stroke in cancer patients has distinct characteristics different from conventional stroke and has higher mortality.

**Patient concerns:**

A 54-year-old female presented to the Department of Otolaryngology with recurrent right nasal bleeding for 4 days. After surgery, the patient experienced 7 different cerebral infarction courses. Finally died of brain herniation.

**Diagnosis:**

The specific abnormal laboratory index is the increase of D-dimer, suggesting the hypercoagulation state. The patient developed multiple cerebral infarction, myocardial injury, renal infarction, splenic infarction, and lower extremity arterial thrombosis, and finally was diagnosed Trousseau syndrome.

**Interventions:**

In the treatment, aspirin and atorvastatin were selected, but it did not work very well. D-dimer were high, we used low molecular weight heparin, and D-dimer decreased significantly.

**Outcomes:**

Finally the patient died of brain herniation.

**Conclusion:**

The raise of D-dimer and typical magnetic resonance imaging manifestation which provides a greater basis for diagnosis. The specific abnormal laboratory index is the increase of D-dimer, which provides direction for treatment and helps to evaluate treatment effect.

## 1. Introduction

The clinical symptoms of Trousseau syndrome are mostly migratory phlebitis, cerebrovascular accident, myocardial infarction, peripheral arterial occlusion, venous thromboembolism, idiopathic deep vein thrombosis, hepatic veno-occlusive disease, and thrombotic thrombocytopenic purpura, multiple organ dysfunction syndrome, and disseminated intravascular coagulation.^[1–3]^ In this case, the patient developed multiple cerebral infarction, myocardial injury, renal infarction, splenic infarction, and lower extremity arterial thrombosis, and finally was diagnosed Trousseau syndrome. Combined with the literature, the specific abnormal laboratory marker was the elevation of the D-D polymers, not only provide a basis for treatment, but also a key indicator of diagnosis. The typical magnetic resonance imaging (MRI) manifestation is the “Three Territory Sign” (TTS: bilateral anterior and posterior circulation acute ischemic diffusion-weighted imaging lesions), as a major diagnostic elements to Trousseau syndrome.

## 2. Case report

**Figure 1.**
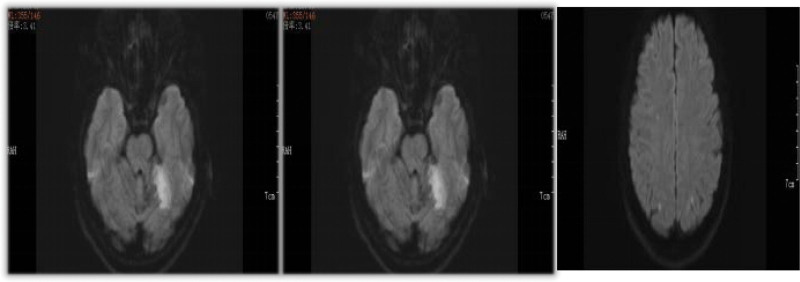
Diffusion-weighted magnetic resonance imaging of the brain showing acute cerebral infarction in the area concerning the left cerebellum and bilateral frontal parietal lobe.

**Figure 2.**
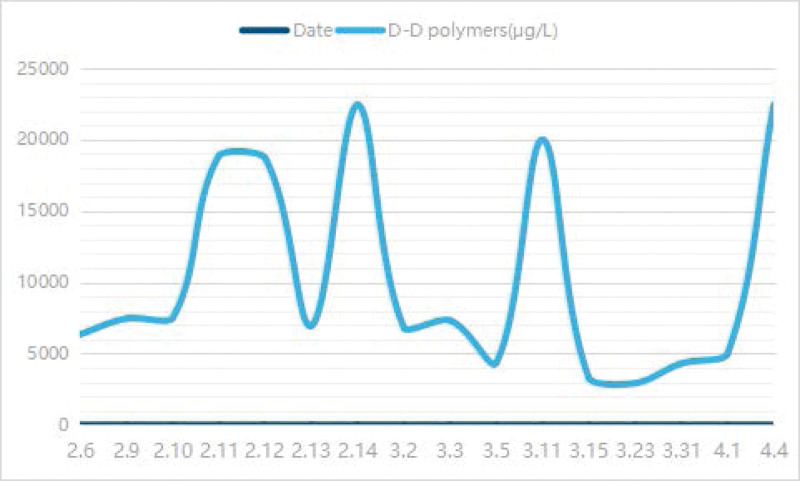
The change of D-Dimer in the course of disease.

A 54-year-old female presented to the Department of Otolaryngology with recurrent right nasal bleeding for 4 days. The patient had no hypertension, diabetes, and no family genetic history. Physical examination showed that the patient’s right nasal septum was severely deviated, and active bleeding was observed in the deep part of the nasal cavity. Surgery was performed and successful. At 7:00 pm on the next day, the patient developed dizziness, nausea, and vomiting several times. The neurological department was consulted, and acute cerebral infarction was considered, which was also confirmed by Magnetic Resonance Examination (Fig. 1), and the patient was transferred to the neurology department for further treatment. During the hospitalization, complete set of tumor, homocysteine, blood sugar, blood pressure, etc were not abnormal, and the cardiac ultrasound, electrocardiogram. Chest computed tomography, artery computed tomography of neck, and magnetic resonance angiography were all normal. Cardiac Troponin I (cTnI) was also elevated to varying degrees, fluctuating between 0.046 and 1.93 ng/mL, suggesting myocardial cell damage. D-dimer were increased also (Fig. 2). In the treatment, aspirin and atorvastatin were selected; D-dimer were high; we used low molecular weight heparin (LMWH), and D-dimer decreased significantly. She was discharged with improved Rankin score of 1.

**Figure 3.**
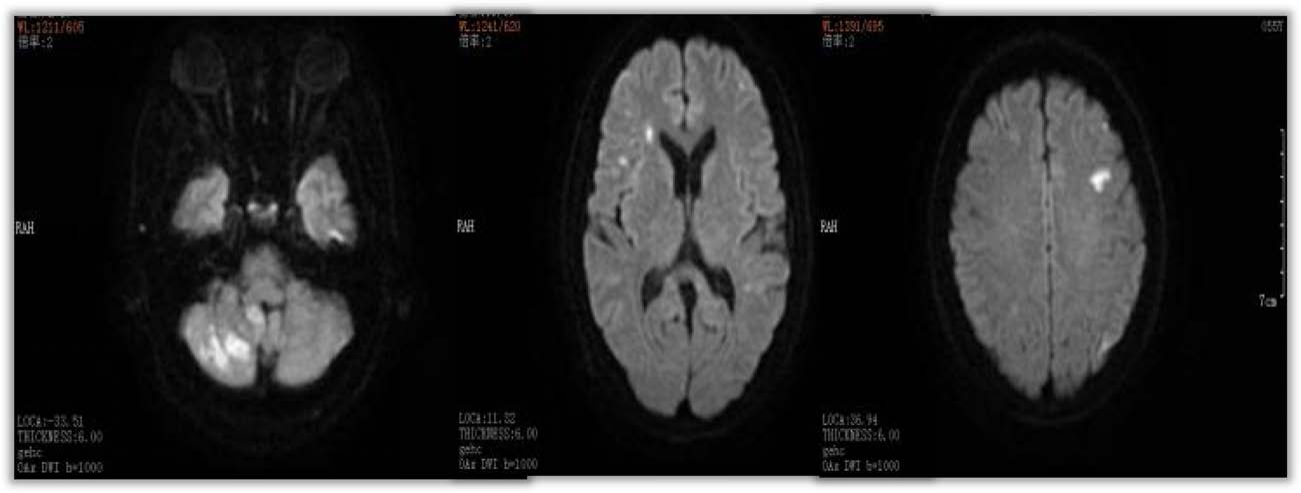
Diffusion-weighted magnetic resonance imaging of the brain showing acute cerebral infarction in the area concerning the right cerebellum and bilateral frontal lobes.

**Figure 4.**
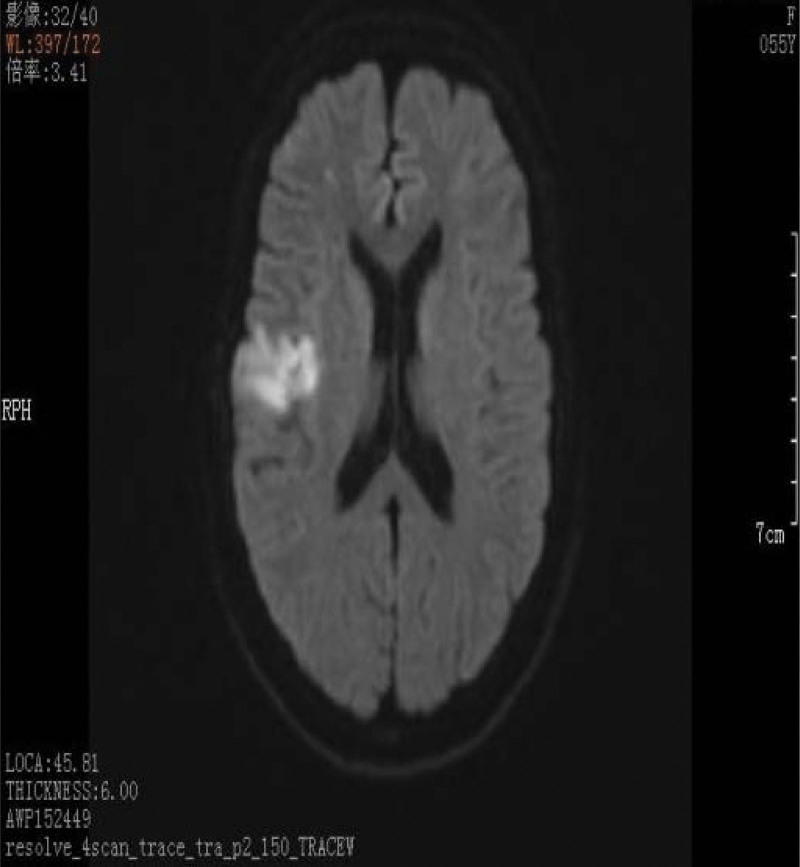
Diffusion-weighted magnetic resonance imaging of the brain showing acute cerebral infarction in the area concerning the right temporal parietal lobe.

**Figure 5.**
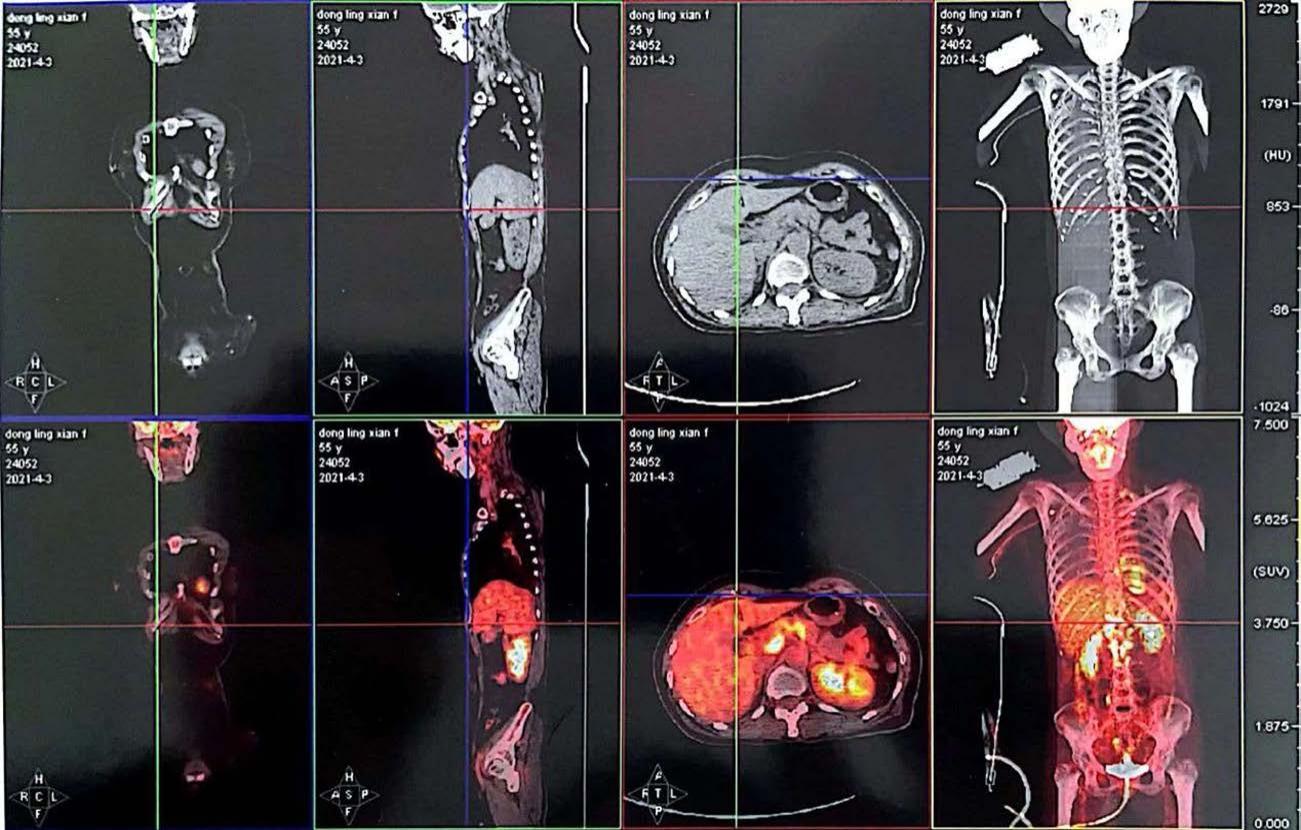
PETCT was suggested that there is abnormal increase of glucose metabolism in the multiple enlarged lymph nodes adjacent to the head of the pancreas (the boundary with the head of the pancreas is unclear), and the possibility of malignancy is highly considered. PETCT = positron emission tomography computer tomography.

We believed that the cause of cerebral infarction was hypercoagulation induced by surgery. The patient experienced similar course for 6 times, which forced us to redetermine the cause of the disease. At 20 days after discharge, the patient developed increased dizziness. The patient had obvious vomiting symptoms and lumbar puncture was normal. Cranial MRI suggested more lesions than before (Fig. 3). The cTnI and D-dimer was still higher (Fig. 2). In view of these 2 abnormal results, the possibility of cardiogenic stroke should be investigated. The foaming test, transesophageal ultrasound, dynamic electrocardiogram, microembolus monitoring, and pulmonary artery examination were all normal. One week after discharge, the patient developed slurred speech and crooked mouth angle, and cranial MRI found new lesions (Fig. 4). LMWH was given, and later transitioned to rivaroxaban. Aphasia occurred during hospitalization, and positron emission tomography computer tomography was suggested that there is abnormal increase of glucose metabolism in the multiple enlarged lymph nodes adjacent to the head of the pancreas (the boundary with the head of the pancreas is unclear), and the possibility of malignancy is highly considered (Fig. 5). Consultation of general surgery considered pancreatic cancer. The reexamination of the full set of tumors showed an upward trend compared with the previous one, among which the cytokeratin 19 fragment was 5.13 ng/mL, the neuron-specific enolase was 31.8 ng/mL, the carbohydrate antigen was 72 to 430.020 KU/L, and the carcinoembryonic antigen was 6.37 ng/mL. During the treatment, the symptoms of right hemiplegia occurred. After anticoagulation and rehabilitation treatment, the patient reappeared with new symptoms, manifested as unresponsiveness. The color Doppler ultrasound of the lower extremity showed arterial embolism in the left lower extremity. The contrast-enhanced computer tomography of the whole abdomen showed infarction of the left kidney and spleen, and multiple enlarged retroperitoneal lymph nodes. The patient eventually developed a massive cerebral infarction and died of brain herniation.

## 3. Discussion

In this case, which was diagnosed Trousseau syndrome finally surgery is a predisposing factor. After the use of low molecular weight heparin, D-Dimer showed an obvious downward trend, and the condition is relatively stable. Studies have confirmed that LMWH is the preferred treatment and prevention method for cancer-related thromboembolic events, and oral anticoagulant drugs have been shown to be ineffective.^[4]^ In the OASIS-CANCER trial, suggested that successful correction of hypercoagulability based on D-dimer values was associated with improved survival and reduced recurrence.^[5]^ This relies mainly on LMWH.

Currently it is believed that the pathogenesis of Trousseau syndrome is caused by a series of overlapping mechanisms. Hypercoagulable state is a main reason. In this state, nonbacterial thrombotic endocarditis is a potential source of thromboembolism and one of the most common causes of ischemic stroke in patients with malignant tumors and also cause primary thrombus.^[6–8]^ Under the complex hypercoagulable mechanism, the incidence of cerebrovascular ischemic events in patients with malignant tumors is as high as 15%. It is considered that the specific abnormal laboratory marker was the elevation of the D-dimer, so the treatment was mainly centered around anticoagulation. At present, studies and various guidelines recommend LMWH. Monitoring the level of D-dimer, which is helpful to evaluate the treatment effects.^[1,2]^

In addition, a retrospective study by Nouh et al^[2]^ suggested the “TTS” (bilateral anterior and posterior circulation acute ischemic diffusion-weighted imaging lesions) as a major contributor to Trousseau syndrome. A evaluation of all presentations of TTS identified 19 different etiologies. In the absence of any identifiable embolic source, acute ischemic cerebral infarction with tumor-associated hypercoagulability (Trousseau syndrome) accounted for 75% of all cases. The study found that TTS were observed 6 times more frequently in malignancy related ischemic strokes than in atrial fibrillation related ischemic strokes.^[3]^

In this case, the patient developed multiple cerebral infarction, myocardial injury, renal infarction, splenic infarction, and lower extremity arterial thrombosis. The specific abnormal laboratory marker was the elevation of the D-D polymers; the typical MRI manifestation is the TTS. Finally was diagnosed Trousseau syndrome.

In conclusion, for patients with acute ischemic stroke, especially when the etiology is not yet clear, with elevated D-dimer aggregates and imaging manifestations of TTS, the underlying mechanism of occult malignancy should be considered. Tumor investigation is required as part of the assessment. The key to secondary prevention in patients considering Trousseau syndrome is the reduction of D-dimer aggregates. D-dimer levels may reflect the severity of a patient’s hypercoagulable state, response to treatment, and subsequent risk of death. Larger randomized prospective trials are needed to further evaluate the characteristics of Trousseau syndrome.

## Author contributions

**Conceptualization:** Xiao Jing Liu.

**Formal analysis:** Ning Yuan Zhang.

**Supervision:** Yu Xiang Liu.

**Writing – original draft:** Xiao Jing Liu.

**Writing – review & editing:** Yu Xiang Liu, Ning Yuan Zhang.
